# Smad3 promotes adverse cardiovascular remodeling and dysfunction in doxorubicin-treated hearts

**DOI:** 10.1152/ajpheart.00312.2022

**Published:** 2022-10-21

**Authors:** Melissa S. Cobb, Shixin Tao, Katherine Shortt, Magdy Girgis, Jeryl Hauptman, Jill Schriewer, Zaphrirah Chin, Edward Dorfman, Kyle Campbell, Daniel P. Heruth, Ralph V. Shohet, Buddhadeb Dawn, Eugene A. Konorev

**Affiliations:** ^1^Department of Basic Sciences, Kansas City University, Kansas City, Missouri; ^2^Ambry Genetics, Department of Advanced Analytics, Aliso Viejo, California; ^3^Department of Internal Medicine, Kirk Kerkorian School of Medicine at UNLV, Las Vegas, Nevada; ^4^The Children’s Mercy Research Institute, Kansas City, Missouri; ^5^Department of Pediatrics, University of Missouri—Kansas City School of Medicine, Kansas City, Missouri; ^6^Department of Medicine, John A. Burns School of Medicine, University of Hawaii, Honolulu, Hawaii

**Keywords:** cardiac remodeling, cardiomyopathy, doxorubicin, endothelium, inflammation

## Abstract

Many anticancer therapies cause serious cardiovascular complications that degrade quality of life and cause early mortality in treated patients. Specifically, doxorubicin is known as an effective anticancer agent that causes cardiomyopathy in treated patients. There has been growing interest in defining the role of endothelial cells in cardiac damage by doxorubicin. We have shown in the present study that endothelial nuclei accumulate more intravenously administered doxorubicin than other cardiac cell types. Doxorubicin enhanced cardiac production of the transforming growth factor-β (TGF-β) ligands and nuclear translocation of phospho-Smad3 in both cultured and in vivo cardiac endothelial cells. To examine the role of the TGF-β/mothers against decapentaplegic homolog 3 (Smad3) pathway in cardiac damage by doxorubicin, we used both *Smad3* shRNA stable endothelial cell lines and Smad3-knockout mice. We demonstrated using endothelial transcriptome analysis that upregulation of the TGF-β and inflammatory cytokine/cytokine receptor pathways, as well as suppression of cell cycle and angiogenesis by doxorubicin, were alleviated in Smad3-deficient endothelial cells. The results of transcriptomic analysis were validated using qPCR, immunoblotting, and ex vivo aortic ring sprouting assays. Similarly, increased cardiac expression of cytokines and chemokines observed in treated wild-type mice was diminished in treated Smad3-knockout animals. We also detected increased end-diastolic diameter and depressed systolic function in doxorubicin-treated wild-type but not Smad3-knockout mice. This work provides evidence for the critical role of the canonical TGF-β/Smad3 pathway in cardiac damage by doxorubicin.

**NEW & NOTEWORTHY** Microvascular endothelial cells in the heart accumulate more intravenously administered doxorubicin than nonendothelial cardiac cell types. The treatment enhanced the TGF-β/Smad3 pathway and elicited endothelial cell senescence and inflammatory responses followed by adverse cardiac remodeling and dysfunction in wild-type but not Smad3-deficient animals. Our study suggests that the TGF-β/Smad3 pathway contributes to the development of doxorubicin cardiomyopathy and the potential value of novel approaches to ameliorate cardiotoxicity by targeting the Smad3 transcription factor.

## INTRODUCTION

Because of advances in early detection, improved therapies, and supportive care, cancer survival rates have increased substantially over the past decades. More people are living after a diagnosis of cancer than ever before, and this cohort now accounts for around 5% of the US population (1). Anticancer treatments used in these patients can cause serious cardiovascular toxicities that degrade quality of life and cause early mortality (2, 3). Of particular concern are doxorubicin and other anthracycline drugs that are used to treat many hematologic and solid tumors (3, 4). Systolic and diastolic dysfunction, left ventricular dilation, and increased plasma cardiac troponin I observed after doxorubicin therapy suggest long-term cardiac damage and adverse remodeling known as delayed doxorubicin cardiomyopathy (5–8). This condition is refractory to conventional therapy and can rapidly progress to heart failure causing high mortality within one year of diagnosis (9). The main approach to limiting cardiac damage has been to cap the cumulative dose of the drug. However, this can lead to suboptimal cancer treatment outcomes, and cardiac abnormalities are sometimes recognized even with moderate doses of doxorubicin (4).

Although cardiomyopathy in treated patients remains a pressing clinical issue, it is critical to establish the mechanisms that account for the often-delayed onset of ventricular dysfunction, the persistence of damage that develops after completion of therapy, and the selective vulnerability of cardiac tissue to doxorubicin. Considerable effort has gone into identifying possible mechanisms of cardiomyopathy (10–13). Although the majority of studies have focused on cardiomyocytes as a primary target of the drug, there is growing interest in defining the role of cardiac vasculature in the toxicity of doxorubicin and other anticancer drugs (14–16). Multiple mechanisms of endothelial cell damage by doxorubicin, including apoptosis, senescence, and breakdown of the endothelial barrier function, have been demonstrated in vitro and in vivo (16–19). Our recent study showed that damage to cultured endothelial cells by doxorubicin is mediated by the activated transforming growth factor-β (TGF-β) pathway (20). In doxorubicin-treated endothelial cells, TGF-β family ligands suppressed endothelial proliferation and formation of vascular structures. These deleterious endothelial effects were alleviated by a pharmacological inhibitor of the TGF-β pathway.

Acting on fibroblasts, cardiomyocytes, and inflammatory cells, TGF-β family members are known to promote myocyte hypertrophy, fibrotic tissue deposition of extracellular matrix components, and changes in ventricular size and geometry (21, 22). The role of this pathway in macrovascular disease, including atherosclerosis, transplant vasculopathy, aortic aneurysm, and coronary restenosis, is now emerging (23–27). Effects of TGF-β on microvascular networks and endothelial cells in particular are diverse and depend on contextual and environmental cues (28). Antiangiogenic effects of TGF-β on developing vasculature, including suppression of endothelial proliferation, migration, and sprouting, have been reported (29–31). Although the effects of TGF-β on developing vascular networks have been well described, most of the vasculature in adult tissues, including cardiac tissue, is largely quiescent. Contribution of the TGF-β pathway to maintenance of quiescent endothelium is not well understood.

The TGF-β family members signal through receptors featuring intrinsic serine-threonine kinase (32) that phosphorylates Smad2 and Smad3 transcription factors (33). As a result of interaction with their DNA-binding partners, Smad proteins induce specific cell type- and context-dependent transcriptional responses (34–37). Despite the critical role of this signaling in cardiac remodeling, no TGF-β pathway-based interventions have been developed to alleviate its deleterious effects on cardiac function. To determine the utility of the TGF-β targeting therapies, its effects on specific cardiac cell types and associated signaling pathways need to be more precisely defined. Since Smad3 occupies a central position within this pathway, we examined the role of this transcription factor in the effects of doxorubicin on cardiac endothelial cells and development of cardiomyopathy. In the present study, we provide evidence that microvascular endothelial cells in the heart accumulate more doxorubicin than nonendothelial cardiac cell types. The treatment elicited endothelial cell senescence and inflammatory responses followed by adverse cardiac remodeling and dysfunction in wild-type but not Smad3-deficient animals. Our data suggest that endothelial cells are a critical target of doxorubicin in the heart and that the TGF-β/Smad3 pathway contributes to the development of doxorubicin cardiomyopathy.

## MATERIALS AND METHODS

### Mice and Doxorubicin Cardiomyopathy Model

Animal experiments were performed in accordance with National Institutes of Health *Guide for the Care and Use of Laboratory Animals* and approved by the Kansas City University, the University of Missouri-Kansas City, and the University of Kansas Medical Center Institutional Animal Care and Use Committees. Smad3-deficient 129-Smad3tm1Par/J mice (38) were purchased from Jackson Laboratories (Stock No. 3451) and bred for this study. Genotyping was performed using the following primers: Smad3 forward primer (5′-TGG ACT TAG GAG ACG GCA GTC C-3′), Smad3 WT-reverse primer (5′-CTT CTG AGA CCC TCC TGA GTA GG-3′), and Smad3 null-reverse primer (5′-CTC TAG AGC GGC CTA CGT TTG G-3′). Male mice were used in this study because of their greater susceptibility to doxorubicin cardiotoxicity (39–41). Doxorubicin was dissolved in sterile saline for injections, and solution was filter sterilized using 0.2-µm filter. Starting at 7 wk of age, mice received one or four weekly tail vein injections of doxorubicin, 5 mg/kg each, or saline in a volume of 150 to 200 µL per injection using 30-G insulin syringes. Intravenous injections were used to model the clinically used route of administration of the drug. Animal weights were monitored weekly beginning with the first injection until the date of euthanasia.

### Reagents and Cell Culture

We used the following antibodies: rabbit monoclonal anti-phospho (p)-Smad3 (Abcam, 1:200 and 1:1,000 dilutions for immunocyto-/immunohistochemistry and immunoblotting, respectively); rabbit monoclonal anti-Smad3 and anti-β-actin (ThermoFisher Scientific, 1:1,000 and 1:10,000 for immunoblotting, respectively); rabbit monoclonal anti-Smad2 (Cell Signaling Technologies, 1:1,000 for immunoblotting); mouse monoclonal anti-CD31 (Dako Agilent Technologies, 1:100 for immunocytochemistry); goat polyclonal anti-IL-6, and rabbit polyclonal pan-specific TGF-β (R&D Systems, 1:40 and 1:200 for immunocytochemistry, respectively); rabbit polyclonal anti-cardiac troponin I (Abcam, 1:100 for immunohistochemistry); FITC-conjugated rat anti-mouse CD31 and APC-conjugated rat anti-mouse CD45 (BD Biosciences, 1:200 for flow cytometry). Lectins [*Griffonia simplicifolia* isolectin B4-biotin, and *Lycopersicon esculentum* (tomato) lectin-biotin] were purchased from Vector Laboratories and used at 1:40 and 1:80 dilutions, respectively.

Secondary reagents used were: HRP-conjugated goat anti-rabbit and horse anti-goat antibodies (Vector, 1:500 for immunohistochemistry); Alexa Fluor 488 and Alexa Fluor 594-conjugated goat anti-rabbit and goat anti-mouse antibodies (ThermoFisher Scientific, 1:500 and 1:1,000 for immunohisto- and immunocytochemistry, respectively); IRDye 680LT goat anti-mouse and IRDye 800CW goat anti-rabbit antibodies (LI-COR Biosciences, 1:5,000 for immunoblotting); and DyLight 488-conjugated streptavidin (Vector, 1:100 for immunohistochemistry). Human recombinant TGF-β1 and mouse recombinant vascular endothelial growth factor (VEGF) were from R&D Systems. Human cardiac microvascular endothelial cells and human umbilical vein endothelial cells were purchased from Lonza and cultured in EGM2-MV and EGM2 complete media, respectively, which were prepared using endothelial basal media (EBM, CC-3156) and SingleQuots supplements (CC-4147 or CC-4176). All experiments with cultured endothelial cells were performed between *passages 6* and *7*. Cells were grown at 37°C and 5% CO_2_ and passaged using 0.05% Trypsin-EDTA that was subsequently neutralized with defined trypsin inhibitor (ThermoFisher Scientific).

### Mouse Aortic Ring Model of Sprouting Angiogenesis

After deep isoflurane anesthesia and thoracotomy, hearts were perfused in situ via the left ventricle with chilled PBS solution containing 10 IU/mL heparin to wash blood out of aortas. The aortas were then ligated with silk suture distal to the aortic arch, and thoracic aorta was excised and transferred to precooled basal Opti-MEM media as described in Ref. (42). After removing periaortic fibrous and adipose tissue, aortas were transversely cut into ∼1-mm rings and incubated in basic Opti-MEM overnight in CO_2_ incubator. Rings were transferred then to the cooled type I rat tail collagen solution (Advanced Biomatrix) in Opti-MEM, adding one ring per well in a 96-well plate. After collagen gelation, aortic rings were incubated in Opti-MEM containing 2.5% FBS and 20 ng/mL mouse recombinant VEGF and treated with 20 nM doxorubicin. To visualize endothelial cell sprouting, aortic rings were fixed in 4% paraformaldehyde and stained using biotinylated IL-B4 followed by DyLight 488-streptavidin conjugate. Sprouting aortic rings were imaged using Cytation 5 Imager (BioTek Instruments) and analyzed with ImageJ software.

### Stable shRNA Cell Lines

*Smad3* and scrambled short hairpin RNAs (shRNAs) were designed using Clontech online algorithm (http://bioinfo.clontech.com/rnaidesigner/oligoDesigner.do). Lenti-X viral particles were generated by recombining the selected shRNA sequences into the pLVX/shRNA1 vector (Takara Bio USA). The selected plasmids were transfected with a packaging mix (Takara Bio USA) into HEK-293T/17 cells. Viral particles were harvested at 48 and 72 h after transfection, clarified using a 0.45-µm filter (MilliporeSigma) and concentrated using Lenti-X concentrator. Viral particles were resuspended in PBS and stored at −80°C. Low-passage endothelial cells were cultured in a small volume of complete media and infected with the viral particles. The transfected cell lines were selected with 2 µg/mL puromycin in complete media for two passages. Stable shRNA cell lines were frozen at passage five.

### Immunoblotting and Quantitative PCR

Endothelial cells were seeded at a density of 0.9–1.5 × 10^4^ cells/cm^2^, treated with 16 nM doxorubicin for 72 h and starved for 4 h using growth factor free media before adding 0.3 ng/mL TGF-β1. Cells were lysed using M-PER extraction buffer with the HALT phosphatase/protease inhibitor cocktail (both by ThermoFisher Scientific) on ice for 15 min. Protein concentrations in prepared extracts were quantified with BCA assay kit (ThermoFisher Scientific). Samples were denatured and reduced using 4X Laemmli sample buffer (Bio-Rad) with β-mercaptoethanol. Equivalent amounts of protein were loaded onto a 4–15% TGX gel (Bio-Rad) and run at 125 V for 60–90 min in 1× Tris/Glycine/SDS buffer (Bio-Rad). The resolved protein was transferred to low fluorescence PVDF membrane using the semidry Trans-Blot Turbo transfer system (Bio-Rad). Membranes were blocked with a 1:1 ratio of SeaBlock (ThermoFisher Scientific) and TBS (pH 7.4) and incubated with primary antibodies in blocking buffer with 0.1% Tween-20 overnight shaking at 4°C. Secondary antibodies with the fluorescent tags 800CW and 680LT (LI-COR Biosciences) were diluted in blocking buffer with 0.1% Tween-20. Membranes were imaged on the Odyssey imaging system using Image Studio software for image capture and analysis (LI-COR Biosciences). The amount of phosphorylated protein per lane was normalized to the total parent protein. The amount of total protein per lane was normalized to β-actin.

To extract total RNA, cells were lysed with TRIzol and the RNA was isolated with the Ambion PureLink RNA Mini kit (ThermoFisher Scientific). Complementary DNA was generated using high-capacity cDNA synthesis kit (ThermoFisher Scientific) and semiquantitative RT-PCR was performed using CFX Connect Real-Time System instrument (Bio-Rad) and human TagMan primer/probe gene expression assays that span exon-exon junctions (ThermoFisher Scientific). The threshold counts were normalized to the relative abundance of HPRT1 or HPRT (in human or mouse cells, respectively) using the comparative CT method. Fold-changes in gene expression were calculated as the fold-change for the experimental groups versus control.

### Echocardiography

Echocardiograms were obtained with a Vevo 2100 Ultrasound System (VisualSonics, Fujifilm) equipped with a high-frequency (18–38 MHz) linear array transducer. Mice were lightly anesthetized with isoflurane (3% for induction and 1–1.5% for maintenance) mixed with 1 L/min O_2_ via a facemask. Hair was removed from the anterior chest using a chemical hair remover, and the animals were placed in the left lateral decubitus position. Body temperature was monitored with a rectal temperature probe and maintained close to 37°C with the use of a heating pad throughout the study. Ultrasound gel was applied to the chest and two-dimensional and M-mode images were acquired from modified parasternal long-axis and short-axis views (43). All images were digitally stored for offline analysis using Vevo LAB software (version 5.5.0, VisualSonics). Systolic and diastolic anatomic measurements were obtained from M-mode images at the midpapillary level. Radial strain was measured in short-axis views and represented as positive curves reflecting increasing myocardial thickness during systole and diminishing wall thickness during diastole and depicting myocardial deformation toward the center of the LV cavity. Analysis was performed by an investigator who was blind to the treatment group allocation and unaware of data from other modalities. Myocardial strain analysis was performed using Vevo Strain software (VisualSonics).

### Tissue Harvesting, Immunohistochemistry, and Immunofluorescence Microscopy

Mice were weighed, injected intraperitoneally with 30 µL heparin (1,000 U/mL), and anesthetized with isoflurane. After medial thoracotomy, mouse hearts were perfused in situ with PBS with 10 U/mL heparin followed by PBS containing 1% paraformaldehyde (PFA), excised and weighed, then fixed in 4% paraformaldehyde in PBS at 4°C overnight. Fixed hearts were processed for paraffin embedding or flash frozen in optimal cutting temperature (OCT) compound cooled in isopentane on dry ice. Frozen tissues were cryosectioned and stored at −80°C until the staining procedures. Paraffin-embedded cardiac sections were deparaffinized, rehydrated, and underwent antigen retrieval in 10 mM citrate buffer (pH 6.0). Cardiac sections and cultured cells were blocked with PBS containing 3% BSA and 0.3% Triton X-100 and incubated with primary antibodies overnight at 4°C. Secondary antibodies conjugated with fluorophores or horseradish peroxidase were used for fluorescence or bright-field imaging, respectively. Endothelial cells were labeled using CD31 antibody, or isolectin B4-biotin or *Lycopersicon esculentum* (tomato) lectin-biotin followed by fluorophore-conjugated streptavidin (44). Nuclei were stained with Hoechst 33342, and slides were mounted with Antifade mounting media. Image acquisition and analysis of fluorescent images were performed using Cytation 5 imaging system with Gen5 3.08 Image Prime software (Biotek Instruments). Bright-field images were acquired using the Cytation 5 system and analyzed using Image-Pro Premier software (Media Cybernetics).

### Detection of Cellular Doxorubicin Accumulation

To evaluate accumulation of doxorubicin in cardiac cells, mice were injected via the tail vein with 5 mg/kg doxorubicin, anesthetized, and then intravenously injected with the vital dye isolectin B4-DyLight 649 (Vector Laboratories, 100 µL of 1 mg/mL solution per injection) to label endothelial cells. Excised hearts were immediately cannulated and briefly perfused via aorta at a rate of 1 mL/min with PBS containing 16 mM KCl and 10 U/mL heparin followed by PBS containing 4% paraformaldehyde. Hearts were then immediately flash frozen in OCT compound cooled with isopentane on dry ice. Cardiac sections were mounted with Antifade media containing 2 µM Hoechst 33342 and imaged immediately.

### Isolation of Mouse Cardiac Endothelial Cells

Cardiac endothelial cells were isolated from mice treated with doxorubicin or saline 9 wk after the last injection, as described in (45) with modifications. Briefly, hearts were perfused in situ with cold PBS-heparin (10 IU/mL) to wash out blood-derived cells, excised, minced on ice, and incubated with collagenase, type 2 (Worthington Biochemical). After collagenase digestion, cell suspension was filtered and centrifuged to pellet cardiac cells and incubated with anti-mouse CD31 conjugated magnetic beads (Miltenyi Biotec). Endothelial cells were separated by passing through a column using QuadroMACS separator (Miltenyi Biotec). To increase purity of the preparation, the eluted cells underwent a second round of selection by passing through another separator column and were then used for isolation of total RNA.

### RNA Sequencing and Transcriptomic Analysis

RNA was isolated from cultured cells or cells isolated from cardiac tissues using the Ambion mirVana RNA Isolation Kit (ThermoFisher Scientific). One microgram of RNA isolated from endothelial cells was converted into cDNA libraries and prepared for sequencing using the Illumina TruSeq RNA sample preparation kit. High output RNA-seq was performed by paired-end (2 × 101) deep sequencing coverage to an average depth of ∼50 million reads with >89% of bases above Q30 using Illumina’s TruSeq technology on the Illumina HiSeq1500. The resulting base calling (.bcl) files were converted to FASTQ files using Illumina’s bcl2fastq v2.17.1.14 software. Sequencing of RNA isolated from mouse cardiac endothelial cell preparations was performed by Novogene Corporation. The Tuxedo Suite pipeline (TopHat v1.3.0/Bowtie v0.12.7/Cufflinks v1.0.3) was used to map RNA-seq reads to the GrCh38 reference genome and to determine transcript assembly and abundance estimations in fragments per kilo base of exon per million fragments mapped (FPKM) according to published procedures (46, 47). Cufflinks and cuffdiff were used to evaluate differential expression, and the per-target *P* values were −Log10 transformed and graphed against Log2 FC (fold-change) of FPKM in a volcano plot in R using ggplot2. Log2 FC values of inf and −inf were omitted from the plot. Significantly upregulated and downregulated targets are shown in red [Log2 FC ≥ 2 or ≤ −2, *p* < 0.05]. Cuffdiff data were filtered to gene_ids with log2 FC >1 or <−1 and a test status of “ok,” yielding 2 to 3 k genes per list. We used http://bioinformatics.psb.ugent.be/webtools/Venn/ to make the Venn diagrams from the filtered cuffdiff gene lists. The same gene lists were submitted for Ingenuity Pathway Analysis (IPA; www.ingenuity.com) to gain insight into the biological relationships and signal transduction cascades altered by experimental treatments. Genes were then filtered by the common targets between the 2 lists for each comparison (scrambled shRNA + TGF-B1 + doxorubicin versus scrambled shRNA + TGF-B1 and Smad3 shRNA + TGF-B1). FPKM values of 0 were converted to 0.5 to recompute log2 FC values of −inf and inf log2 FC into an approximated graphable value. Sample clustering was performed on log2 transformed expression fold-changes with the R pheatmap package, using default parameters (Euclidian distance and “complete” clustering method) to visualize the differential gene expression across samples. IPA upstream regulator analysis was used to identify common upstream regulators and analyze differentially expressed transcripts downstream of them. The tool then generated associated *Z*-scores and *P* values of overlap that imparted statistical significance and directionality (up- or downregulation) to each upstream regulator (48). For pathway-specific heatmaps, the gene FPKM calculated in Cufflinks were extracted from the sample-level data and used to calculate log2 FC FPKM.

### Statistical Analysis

Statistical analyses were performed using GraphPad Prism (version 9.1.2) and SigmaPlot (version 13.0) software. Experimental data were analyzed initially using the Shapiro-Wilk normality test. Normally distributed data were subjected to parametric tests, including *t* test or analysis of variance (ANOVA). Unpaired *t* test was used to perform comparisons between two experimental groups. When more than two experimental groups within a data set were to be compared, one- or two-way ANOVA followed by either Sidak’s or Tukey’s correction tests were used, as suggested by statistical software. Results are expressed as means ± SD. A *P* < 0.05 was considered statistically significant.

## RESULTS

### Cardiac Microvascular Endothelium Accumulates Increased Amounts of Intravenously Administered Doxorubicin

**Figure 1.**
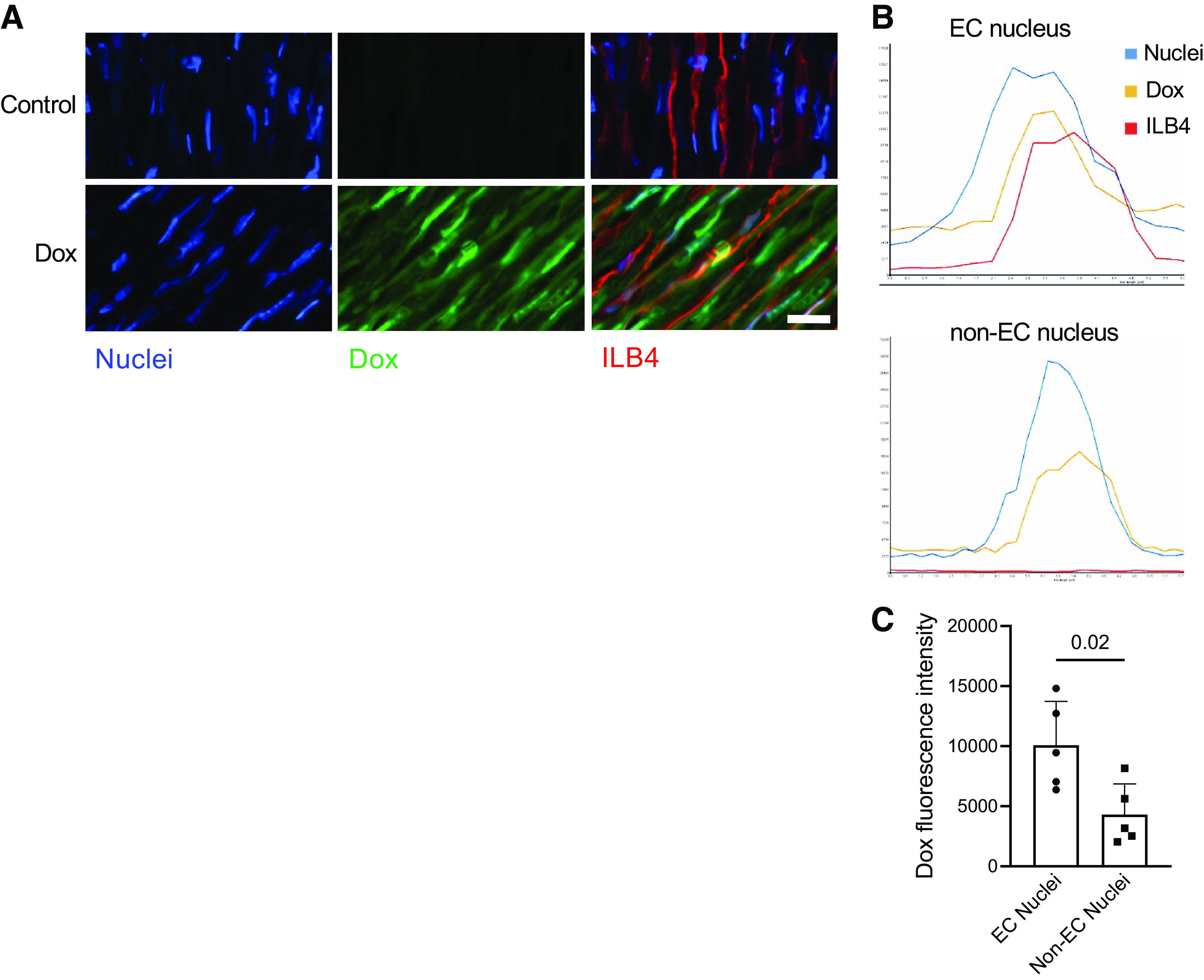
Accumulation of doxorubicin (Dox) in cardiac endothelial cells of treated mice. Mice were intravenously injected with 5 mg/kg doxorubicin under general anesthesia with isoflurane. Heart tissues were harvested 1 h after the injection. Isolectin B4-DyLight 649 conjugate (ILB4) was intravenously injected 5 min before tissue harvesting to label endothelial cells (ECs). *A*: accumulation of doxorubicin in endothelial and non-endothelial nuclei 1 h after its intravenous injection. Intrinsic fluorescence of doxorubicin was pseudocolored green. Control mice did not receive doxorubicin. Nuclei were stained with 2 µM Hoechst 33342, whereas endothelial cells were identified using the ILB4 conjugate. Scale bar = 20 µm. *B*: fluorescence intensity profiles of endothelial cell (EC) and non-endothelial cell (non-EC) nuclei. Endothelial cells were identified using the ILB4 conjugate (shown in red); nuclei were identified using Hoechst 33342 dye (shown in blue). *C*: analysis of doxorubicin accumulation in endothelial vs. non-endothelial nuclei 1 h after doxorubicin injection. Twenty nuclei were analyzed per heart and averaged to present the results of *n* = 5 independent experiments. The *P* value for the unpaired two-tailed *t* test is shown.

Doxorubicin is administered intravenously in the clinical setting and is known to rapidly distribute from the vascular compartment. Endothelial cells are positioned at the interface between tissues and systemic circulation and are exposed to high drug concentrations. We have used intrinsic fluorescence of the doxorubicin molecule to assess its accumulation in endothelial and non-endothelial cardiac cells. Endothelial cells were labeled using fluorophore conjugated lectin ILB4 that was injected intravenously before cardiac tissue harvesting (Fig. 1*A*). As a DNA intercalating agent, doxorubicin is known to accumulate in the nuclei (Fig. 1*B*). Nuclear accumulation of the drug reached maximal values 1 h after the injection, and we detected markedly increased amounts of doxorubicin in endothelial nuclei at this time point, as compared with non-endothelial cardiac cell nuclei (Fig. 1*C*). Thus, although doxorubicin may have multiple targets in treated hearts, we focused in this study on endothelial cells because this cell type accumulates increased amounts of the intravenously administered drug and its cardiovascular toxicity is steeply dose dependent (8, 49).

### Doxorubicin Augments the Endothelial Responses to TGF-β1

**Figure 2.**
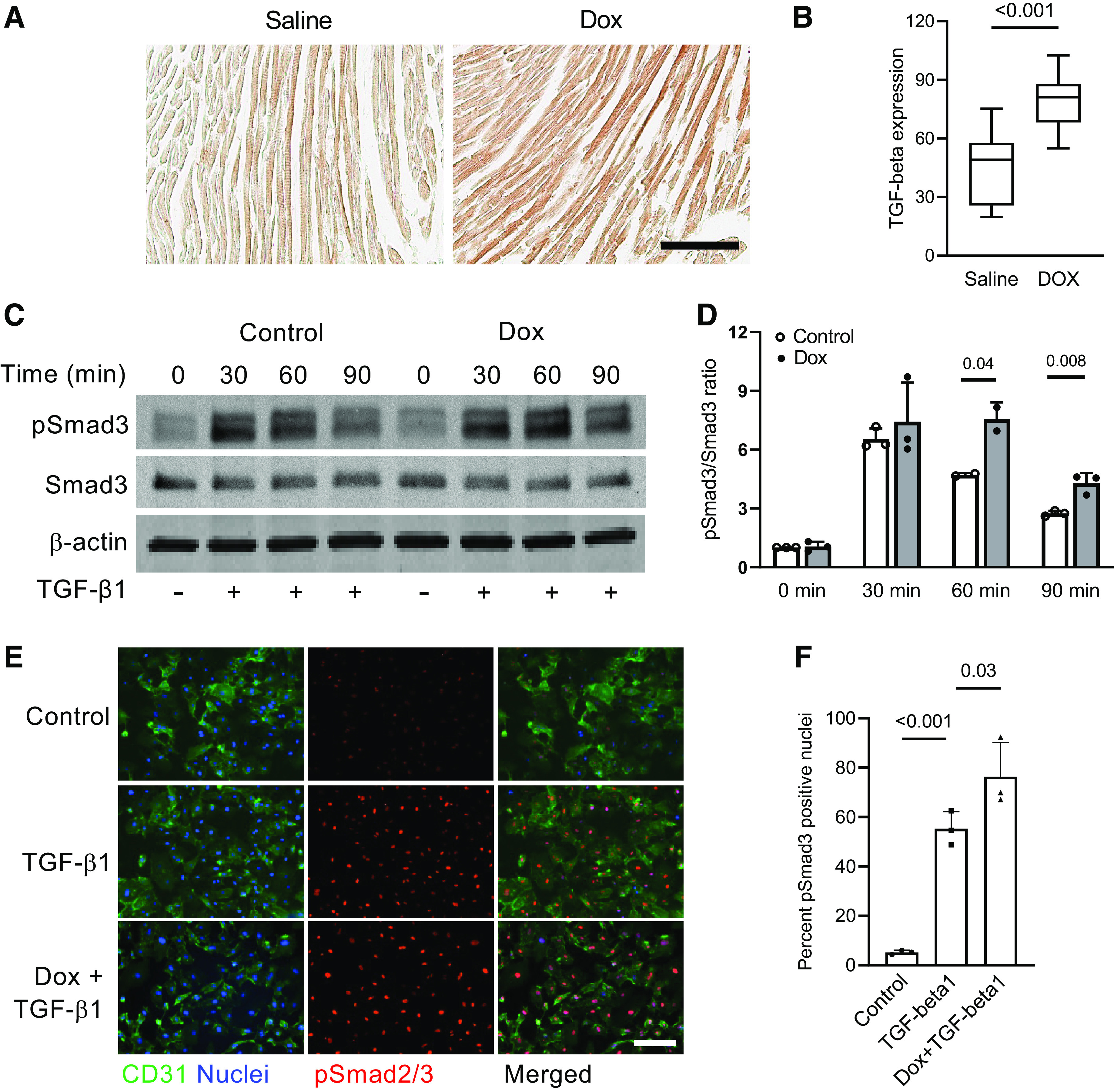
Doxorubicin enhances activity of the canonical TGF-β pathway in endothelial cells. *A*: mice were intravenously injected with doxorubicin (5 mg/kg) or saline, and heart tissues were harvested 24 h after the injections and processed for immunohistochemical staining. Cardiac sections were stained with the TGF-β pan-specific antibody and analyzed using color bright-field microscopy. Scale bar = 100 µm. *B*: TGF-β expression in cardiac tissues was measured by density of horseradish peroxidase substrate 3,3′-diaminobenzidine (DAB) deposits. *n* = 3 hearts per group. Eight to ten randomly selected fields per heart were analyzed. *P* value for the unpaired two-tailed *t* test is shown. *C*: human cardiac microvascular endothelial cells were pretreated with 16 nM doxorubicin (Dox) for 72 h, starved for 4 h, and incubated with 0.3 ng/mL TGF-β1 for 0 to 90 min. Protein extracts were then processed according to the immunoblotting protocol and incubated with the indicated antibodies. pSmad3, phospho-Smad3. *D*: pSmad3 band intensity in cells lysed at the indicated time points after treatment with TGF-β1 is presented as a ratio of phospho- to total Smad3. Results of 3 independent experiments are shown. *P* values for the unpaired two-tailed *t* tests are shown. *E*: increased pSmad2/3 nuclear translocation response to TGF-β1 in endothelial cells pretreated with 16 nM Dox for 72 h, starved for 4 h, and treated with 0.3 ng/mL TGF-β1 for 1 h. Endothelial cells were labeled using CD31 antibody, and nuclei were stained with Hoechst 33342. Scale bar = 200 µm. *F*: quantification of pSmad2/3-positive nuclei (as percentage of total nuclei) in endothelial cells treated with Dox and/or TGF-β1. Results of 3 independent experiments are shown. *P* values for the one-way ANOVA tests are shown.

We have previously detected increased expression of the TGF-β family members in cultured cardiac endothelial cells treated with doxorubicin (20) and sought to evaluate their expression in hearts of doxorubicin-treated mice. Using the TGF-β pan-specific antibody, we detected increased abundance of the TGF-β isoforms in cardiac tissues of doxorubicin-treated mice 24 h after a single injection of the drug (Fig. 2, *A* and *B*). Thus, cardiac endothelial cells are exposed to doxorubicin in the presence of elevated concentrations of the TGF-β ligands. To recreate the in vivo conditions, human endothelial cells were pretreated with doxorubicin and subsequently exposed to TGF-β1 to examine activation of the canonical pathway. We used the same dose of doxorubicin as in the previous study (20) to determine if the endothelial response to TGF-β1 was enhanced by pretreatment with doxorubicin. Doxorubicin pretreatment did not modify the cellular Smad3 phosphorylation response to TGF-β1 after the 30-min incubation (Fig. 2, *C* and *D*). It is noteworthy however that doxorubicin pretreatment increased cellular phospho-Smad3 accumulation at later time points to prolong activation of the TGF-β canonical pathway. We then examined nuclear translocation of phospho-Smad2/3 in cultured endothelial cells exposed to TGF-β1 for 1 h. As shown in Fig. 2, *E* and *F*, phospho-Smad2/3 nuclear translocation after TGF-β1 treatment was increased in doxorubicin-pretreated endothelial cell cultures. Thus, these studies have suggested that, in addition to being exposed to elevated TGF-β concentrations in doxorubicin-treated mice, cardiac endothelial cells exhibit enhanced activation of the canonical pathway in response to a given concentration of TGF-β1.

### Smad3-Dependent Acute Cardiovascular Effects of Doxorubicin

**Figure 3.**
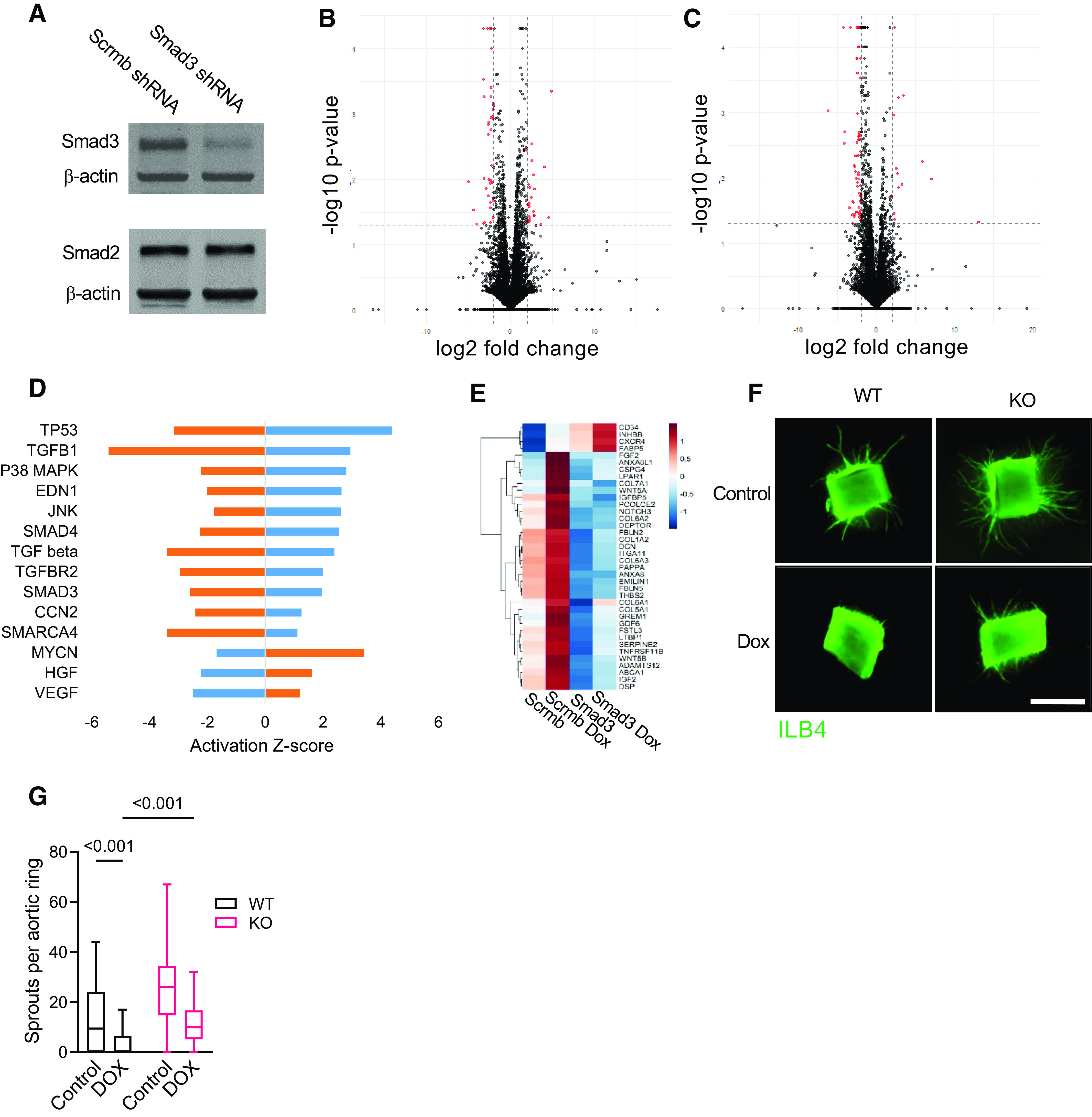
Smad3 deficiency suppresses the TGF-β pathway and promotes sprouting by doxorubicin (Dox)-treated mouse aortic ring explants. *A*: Smad2 and Smad3 expression in Scramble shRNA (Scrmb) and Smad3 shRNA (Smad3) human cardiac microvascular endothelial cell lines. Cell protein lysates were processed according to the immunoblotting protocol and incubated with the indicated antibodies. A representative immunoblot (of 3 independent experiments performed) is shown. *B* and *C*: human cardiac microvascular endothelial cells were pretreated with 16 nM doxorubicin (Dox) for 72 h, starved for 4 h in growth factor-free endothelial basal media, and treated with 0.3 ng/mL TGF-β1 for 16 h. Total RNA was isolated and sequenced, and transcriptomic analysis was performed. Volcano plots for the Scrmb Dox vs. Scrmb (*B*) and Smad3 Dox vs. Scrmb Dox (*C*) comparisons are shown. *D*: activation Z-scores for the upstream regulators identified using Scrmb Dox vs. Scrmb (in blue) and Smad3 Dox vs. Scrmb Dox (in orange) pairwise comparisons analysis. *P* < 0.05 for all presented Z-scores. *E*: heatmap of the TGF-β1 pathway-related transcripts in untreated and doxorubicin-treated scrambled shRNA and Smad3 shRNA endothelial cell lines. Significantly upregulated and downregulated genes for which TGF-β1 is an upstream regulator are indicated in red and blue, respectively. *F*: sprouting of aortic ring explants from wild-type (WT) and Smad3-knockout (KO) mice in collagen matrix visualized using isolectin B4 (ILB4) staining. Scale bar = 1,000 µm. *G*: quantification of aortic ring sprouting. Results of 6 independent experiments are presented with 4 rings per experimental group each. *P* values for the two-way ANOVA tests are shown.

To address the role of Smad3 in endothelial responses to doxorubicin, we created a stable Smad3-deficient cell line. Human cardiac endothelial cells transduced with the *Smad3* shRNA expressing lentivirus showed a marked knockdown of the Smad3 protein (Fig. 3*A*). Importantly, expression of Smad2, a homologous transcription factor protein with 92% amino acid sequence similarity, was not reduced. We then performed transcriptomic analysis to further examine responses to TGF-β1 in doxorubicin-pretreated control and Smad3-deficient endothelial cells. Specifically, we compared doxorubicin-treated versus untreated scrambled shRNA (Fig. 3*B*), and treated Smad3 shRNA versus treated scrambled shRNA (Fig. 3*C*) endothelial transcriptomes to identify differentially expressed genes in these pairwise comparisons and subsequently conduct Ingenuity Pathway Analysis (IPA). As with the Smad3 phosphorylation and nuclear translocation studies, we found enhanced response of endothelial cells pretreated with doxorubicin to TGF-β1 treatment (Fig. 3, *D* and *E*). We also saw that upregulation of the TGF-β pathway by doxorubicin was effectively abrogated in the Smad3-deficient endothelial cells. Analysis of the TGF-β pathway-related genes using semiquantitative PCR corroborated the results of the RNA sequencing (Supplemental Fig. S1; all Supplemental material is available at https://doi.org/10.6084/m9.figshare.21202964). Pathway analysis revealed upregulation of antiproliferative, chromatin remodeling, and profibrotic factors in doxorubicin-treated control but not Smad3-deficient endothelial cells (Fig. 3*D*). Importantly, transcriptomic data suggested suppression of angiogenic pathways by doxorubicin, exemplified by vascular endothelial growth (VEGF) and hepatocyte growth (HGF) factors, and these effects were alleviated in Smad3-deficient cells. We used mouse aortic ring model to examine the role of Smad3 in suppression of angiogenesis by doxorubicin. Embedded in collagen matrix aortic ring explants from wild-type and Smad3-knockout mice were cultured in the presence of VEGF (Fig. 3*F*). Exposure of wild-type explants to doxorubicin profoundly inhibited sprouting angiogenesis but aortic rings from Smad3-knockout animals were more responsive to the angiogenic action of VEGF (Fig. 3*G*).

**Figure 4.**
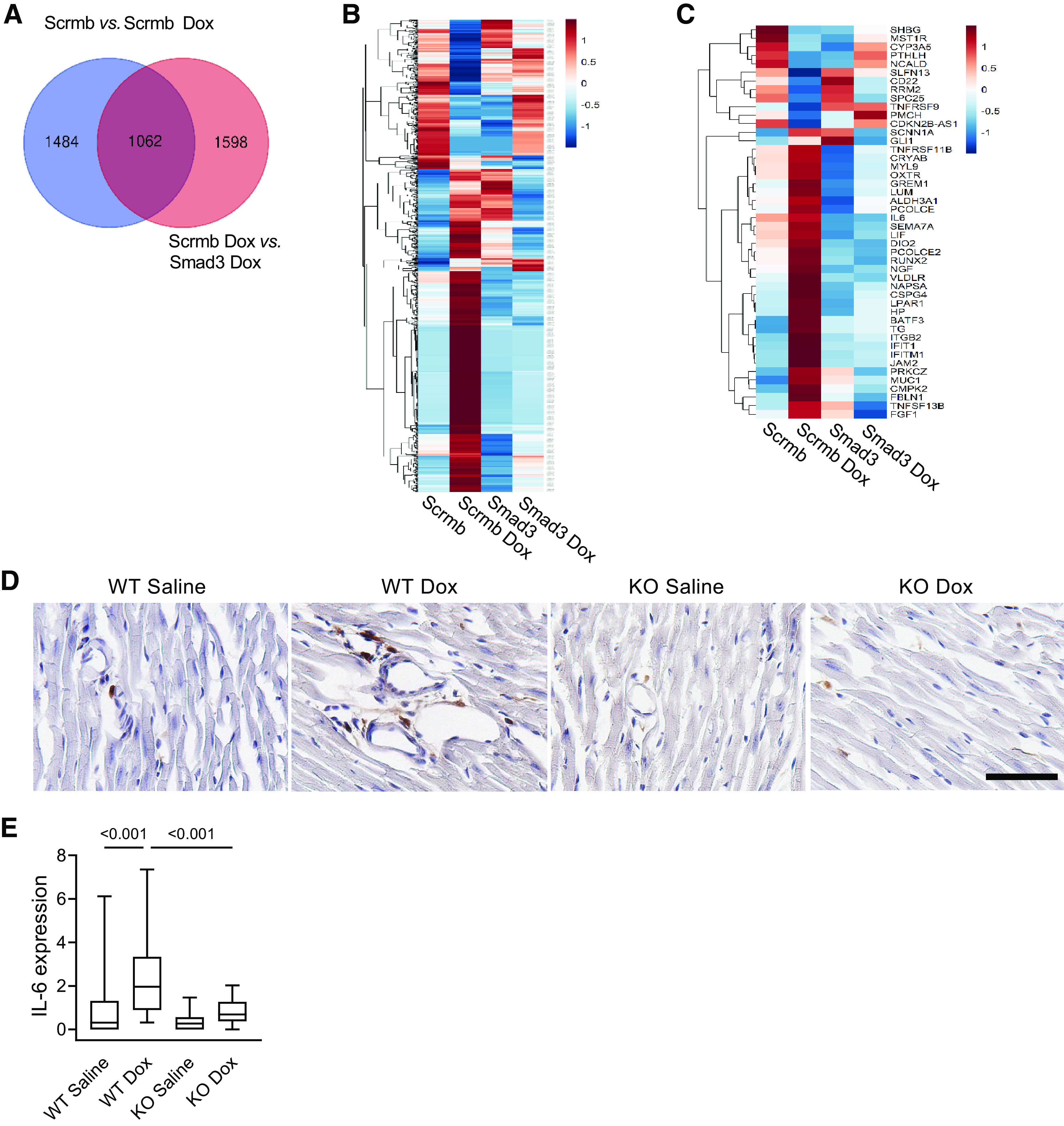
Cardiac inflammatory response to doxorubicin is mediated by Smad3. Human cardiac microvascular endothelial cells were pretreated with 16 nM doxorubicin (Dox) for 72 h, starved for 4 h in growth factor-free endothelial basal media, and treated with 0.3 ng/mL TGF-β1 for 16 h. *A*: Venn diagram quantifying genes overlapping for comparisons shown in Fig. 3, *B* and *C* (in blue and orange, respectively). One thousand and sixty-two genes were significantly differentially expressed and shared between the 2 comparisons. *B*: heatmap of significantly differentially expressed 1,062 transcripts in response to TGF-β1 in pretreated with Dox Scrmb shRNA and Smad3 shRNA endothelial cell lines. *C*: heatmap of significant cytokine signaling-related gene expression in response to TGF-β1 in pretreated with Dox Scrmb shRNA and Smad3 shRNA endothelial cell lines. *D*: IL-6 expression in cardiac tissues of wild-type (WT) and Smad3-knockout (KO) mice 48 h after intravenous injections of 5 mg/kg doxorubicin (Dox) or saline. Cardiac sections were stained using anti-IL-6 antibodies and counterstained with hematoxylin. Scale bar, 50 µm. *E*: quantitative analysis of IL-6 expression in cardiac tissues of treated mice. *n* = 3–6 animals per experimental group, with 6–10 randomly selected fields of view analyzed per heart. *P* values for the two-way ANOVA tests are shown.

Further analysis of differentially expressed transcripts presented in Fig. 3, *B* and *C* yielded 1,062 overlapping differentially expressed genes (Fig. 4*A*). We find that the endothelial transcriptional response to doxorubicin, plotted in the heatmap format in Fig. 4*B*, was largely abolished in *Smad3* shRNA-deficient cells, and their overall gene expression pattern appears to be similar to untreated cells. Results of the IPA upstream regulator analysis for this group of overlapping differentially expressed transcripts is presented in Table 1. Cytokines, cytokine receptors, and transcription factors operating in cytokine signal transduction pathways were activated by doxorubicin and consistently downregulated by Smad3 deficiency. These findings (Fig. 4*C*) support the results of the IPA upstream regulator analysis that activation of the proinflammatory cytokine pathways by doxorubicin in endothelial cells is mediated by Smad3. Analysis of cytokine-related transcripts using semiquantitative PCR corroborated the results of RNA sequencing (Supplemental Fig. S1). We further examined doxorubicin-induced cardiac inflammation. A single injection of doxorubicin significantly increased IL-6 expression in myocardial tissue of wild-type mice (Fig. 4, *D* and *E*), and upregulated IL-6 expression was completely abolished in treated Smad3-knockout animals. Thus, doxorubicin elicits profibrotic, antiangiogenic, and inflammatory responses in both cultured human cardiac endothelial cells and mouse hearts, and Smad3 deficiency markedly reduces these effects in both of these settings.

**Table 1.** Upstream regulator analysis of the differentially expressed overlapping genes: effects of doxorubicin pretreatment on the transcriptional responses to TGF-β1 in Smad3-deficient endothelial cells

Upstream Regulator	Molecule Type	Predicted Activation State	Activation Z-Score	*P* Value of Overlap
IL6	Cytokine	Inhibited	−2.264	0.0003
IL1 (group)	Cytokine group	Inhibited	−2.405	0.0007
IFNG	Cytokine	Inhibited	−2.305	0.0039
CCR2	Cytokine receptor	Inhibited	−2.0	0.0144
IL1A	Cytokine	Inhibited	−2.395	0.0171
RELA	Transcription regulator	Inhibited	−1.492	0.0005
TGFB1	Growth factor	Inhibited	−1.628	0.0012
IL1B	Cytokine	Inhibited	−1.556	0.0027
TNF	Cytokine	Inhibited	−1.868	0.0107
STAT1	Transcription regulator	Inhibited	−1.474	0.0317

Presented in Fig. 4, *B* and *C*, 1,062 significantly differentially expressed genes overlapping for 2 comparisons underwent IPA upstream regulator analysis. Activation *Z*-scores and corresponding *P* values of overlap for the comparison of Scrmb shRNA vs. Smad3 shRNA human endothelial cell cultures pretreated with 16 nM doxorubicin and subsequently treated with 0.3 ng/mL TGF-β1 are shown. The activation *Z*-scores predict the activation state of an upstream regulator using the transcript expression patterns of the molecules downstream of the upstream regulator. The overlap *P* value is calculated by the Ingenuity Pathway Analysis software to estimate a statistically significant overlap between the data set genes and the genes that are regulated by an upstream regulator. CCR2, C-C chemokine receptor type 2; IFNG, interferon-γ; IL1, interleukin-1; IL1A, interleukin-1α; IL1B, interleukin-1β; IL6, interleukin 6; RELA, RELA proto-oncogene, NF-κβ subunit; STAT1, signal transducer and activator of transcription 1; TGFB1, transforming growth factor β1; TNF, tumor necrosis factor.

### Cardiovascular Remodeling after Doxorubicin Treatment Is Smad3 Dependent

**Figure 5.**
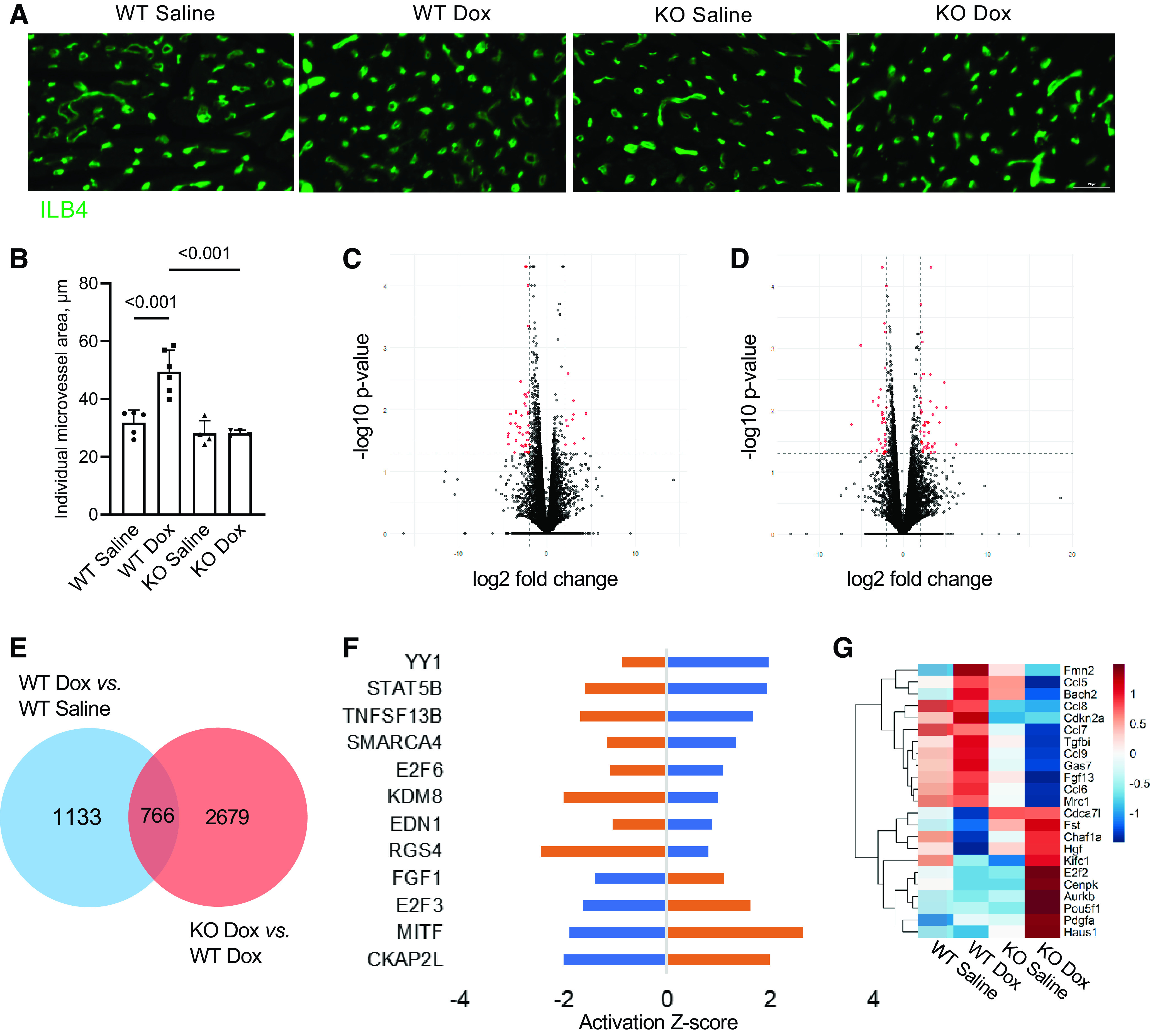
The role of Smad3 in cardiac microvascular remodeling after completion of doxorubicin therapy. Wild-type (WT) and Smad3-knockout (KO) mice were intravenously injected with 4 weekly injections of doxorubicin (Dox, 5 mg/kg each, for a total dose of 20 mg/kg) or saline. Animals were euthanized 9 wk after the last injection of the drug or vehicle. *A*: cardiac sections from treated mice were stained with ILB4 to visualize microvasculature. Scale bar = 20 µm. *B*: quantification of individual microvessel area. *n* = 4 to 6 animals per treatment group. *P* value for the one-way ANOVA test is shown. *C* and *D*: transcriptomic analysis was performed on endothelial cells isolated from hearts of treated mice 9 wk after the last injection. Volcano plots for the WT Dox vs. WT saline (*C*) and Smad3 KO Dox vs. WT Dox (*D*) comparisons. *E*: Venn diagram quantifying genes overlapping for comparisons shown in *C* and *D* (in blue and orange, respectively). Seven hundred and sixty-six genes were significantly differentially expressed and shared between the 2 comparisons. *F*: activation Z-scores for the upstream regulators identified using overlapping differentially expressed genes analysis for the WT Dox vs. WT saline (in blue) and Smad3 KO Dox vs. WT Dox (in orange) comparisons. *P* < 0.05 for all presented Z-scores. *G*: heatmap of significant CDKN2A upstream regulator-related gene expression changes in endothelial cells from treated hearts.

Doxorubicin cardiotoxicity often presents clinically as a delayed cardiomyopathy that develops after completion of therapy. To examine the role of Smad3 in delayed cardiovascular consequences, we performed four weekly injections of doxorubicin and evaluated its cardiac effects 9 wk after the last injection. Although there was no difference in microvascular density between saline and doxorubicin-treated groups (data not shown), we detected increased individual microvessel area in doxorubicin-treated wild-type hearts, as compared with saline controls (Fig. 5, *A* and *B*). This may indicate either abnormal dilation of cardiac microvessels or significantly enlarged endothelial cells similar to the reported bone endothelial cell alterations induced by irradiation or administration of the chemotherapeutic drug 5-fluorouracil (50). No microvascular remodeling occurred in cardiac tissues of Smad3-knockout animals as a result of doxorubicin treatment. We then used highly enriched endothelial cell preparations, characterized in Supplemental Fig. S2, to examine endothelial transcriptomes of wild-type and Smad3-knockout mice after completion of doxorubicin therapy. We subsequently performed pairwise comparisons of wild-type doxorubicin- versus wild-type saline-treated (Fig. 5*C*) and knockout doxorubicin- versus wild-type doxorubicin-treated (Fig. 5*D*) mouse cardiac endothelial cells. IPA upstream regulator analysis of overlapping differentially expressed genes highlighted upregulation of cell cycle arrest (E2F6, FBXW7, CKAP2L, E2F3, FGF1), chromatin remodeling (KDM8, YY1, SMARCA4), and inflammatory (TNFSF13B, EDN1, STAT5B) pathways in wild-type animals that were pretreated with doxorubicin (Fig. 5, *E* and *F*). These results support earlier findings of endothelial senescence and chronic cardiac inflammation after a single dose of doxorubicin (17). That study also reported increased promoter activity of the senescence associated gene *Cdkn2a* encoding cyclin-dependent kinase inhibitor p16^INK4a^ in tissues of doxorubicin-treated mice. We performed upstream regulator analysis of the mouse cardiac endothelial transcriptome to characterize the CDKN2A transcriptional network in doxorubicin-treated wild-type and Smad3-knockout mice. As presented in Fig. 5*G*, doxorubicin decreased expression of genes encoding angiogenic growth factor and factors involved in DNA replication and regulation of mitosis (*E2f2, Hgf, Haus1, Chaf1a*) while increasing abundance of chemokine and p53 target transcripts (*Ccl5, Ccl6, Ccl9, Gas7*). These doxorubicin-induced changes were reduced in endothelial cells from Smad3-knockout animals. The results of the transcriptomic analysis were confirmed using real-time PCR (Supplemental Fig. S3).

**Figure 6.**
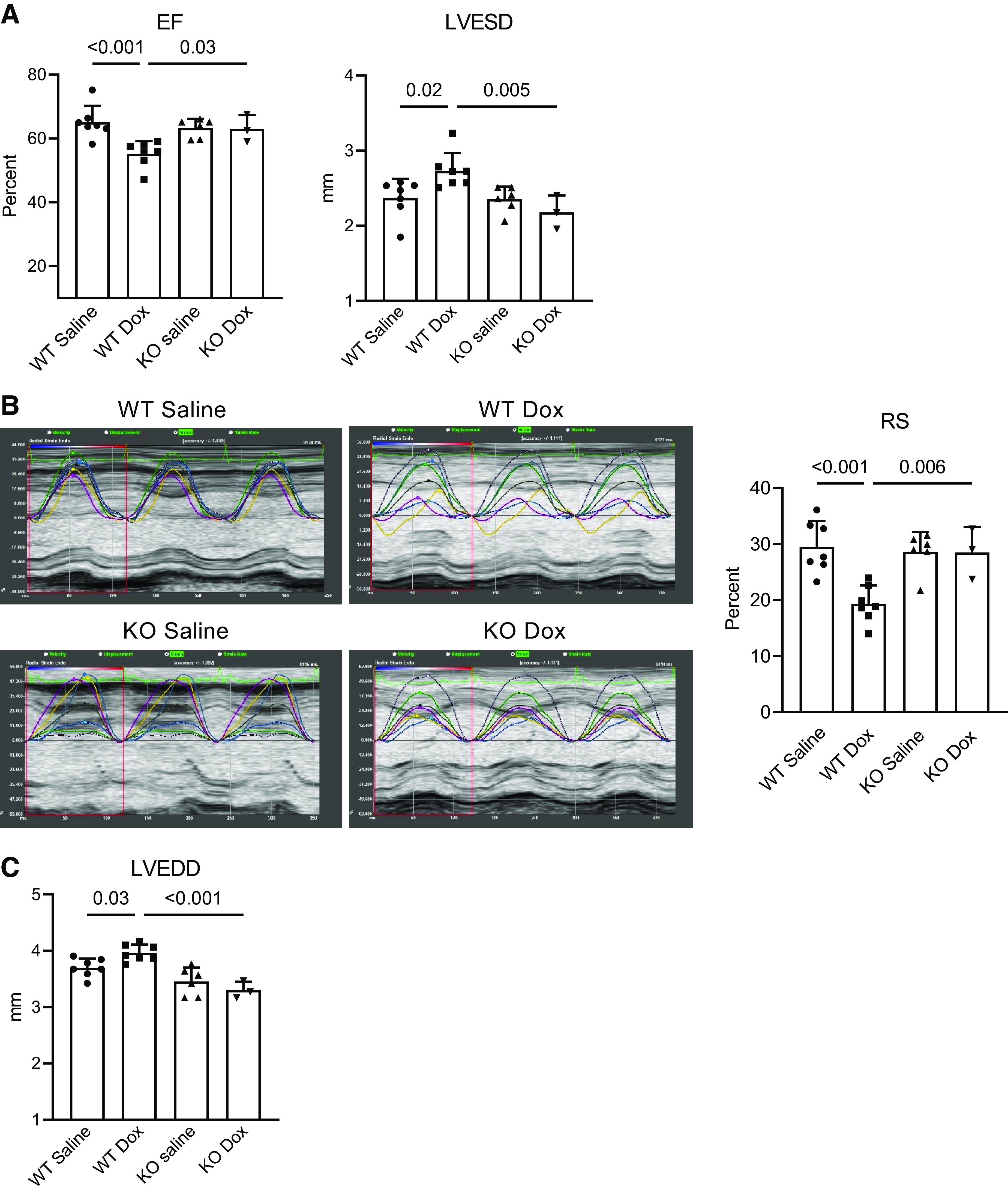
Smad3 deficiency prevents contractile decline and adverse cardiac remodeling in doxorubicin-treated hearts. Wild-type (WT) and Smad3-knockout (KO) mice were intravenously injected with four weekly injections of doxorubicin (Dox, 5 mg/kg each, for a total dose of 20 mg/kg) or saline, and underwent transthoracic echocardiography under light anesthesia with isoflurane 9 wk after the last injection. Experiments on three to seven animals per group were performed. *P* values for the one-way ANOVA tests are shown. *A*: left ventricular systolic function was assessed using ejection fraction (EF) and end-systolic diameter (LVESD). *B*: global radial strain (RS) was examined as an additional measure of left ventricular systolic function. The representative images on the left show separate strain curves generated for each of the six standard myocardial regions, with each cardiac segment represented with a designated color, and the seventh line (black) denoting average strain for the heart at each time point. Quantitative RS analysis is presented in the right panel. *C*: cardiac remodeling was evaluated using left ventricular end-diastolic diameter (LVEDD).

In addition to microvascular remodeling, left ventricular (LV) ejection fraction decreased and LV end-systolic diameter increased in doxorubicin-treated wild-type mice but not in Smad3-knockout animals, indicating that Smad3 deficiency prevented doxorubicin-induced LV systolic dysfunction (Fig. 6*A*). Consistent with these observations, LV global radial strain was markedly decreased in wild-type mice treated with the drug; this adverse effect was also prevented by Smad3 deficiency (Fig. 6*B*). Importantly, LV end-diastolic diameter increased significantly in doxorubicin-treated wild-type hearts, indicating LV dilation and adverse remodeling, also seen in doxorubicin-treated patients who develop cardiomyopathy (Fig. 6*C*). LV dilation was absent in treated Smad3-knockout animals. Together, these results demonstrate adverse cardiovascular remodeling and decline in systolic function in doxorubicin-treated wild-type but not Smad3-knockout mice.

## DISCUSSION

Increased levels and activity of the TGF-β superfamily members, including TGF-β and activin isoforms, and myostatin, have been reported in patients with cardiomyopathy (51–53). Levels of these proteins were positively correlated with the severity of the condition and progression to heart failure (54, 55). Likewise, studies using animal models of heart disease, including myocardial ischemia-reperfusion, aortic constriction, rapid ventricular pacing, and catecholamine infusion, have demonstrated increased activity of the TGF-β pathway (56–60). Suppression of the pathway using small molecular weight inhibitors, neutralizing antibodies and knockout strategies ameliorated ventricular remodeling, as well as systolic and diastolic dysfunction (59, 61–64). These studies have suggested that TGF-β and certain other members of the TGF-β superfamily are involved in cardiac disease progression, and that results of the present study may have broader implications beyond doxorubicin cardiomyopathy. Increase in plasma TGF-β levels has also been detected after anticancer treatments, including doxorubicin, other chemotherapeutic drugs, and radiation (65–67). Furthermore, elevation in plasma TGF-β1 concentration in this setting strongly correlated with the risk of developing posttreatment complications (66, 68). Suppression of the pathway using a TGF-β receptor type I kinase inhibitor alleviated doxorubicin-induced renal vascular damage and nephropathy (69, 70). These data implicated the TGF-β pathway in doxorubicin nephrotoxicity but the specific signaling mechanisms were not determined in these studies.

### Smad3-Mediated Endothelial Responses to Doxorubicin

The risk of cardiovascular complications from doxorubicin is highly correlated with cumulative dose (8, 49). Whereas the doses of the drug in the clinical setting are determined using patients’ body surface area, ratio of body surface area to body mass and the rates of doxorubicin metabolism are vastly different in mice, as compared with humans. The dose of 5 mg/kg used in the present study was reported by others (71) to steadily reduce breast tumor volumes in mice and therefore regarded as clinically relevant. We found that at this dose, cardiac endothelial cells had higher nuclear concentrations of doxorubicin compared with other cells, and thus likely constitute a target of doxorubicin in the heart. Endothelial cells account for over 60% of non-myocyte cells and are the most abundant cell type in the heart (72). Several recent studies have also emphasized an important role of endothelial cells in development of doxorubicin cardiomyopathy (19, 71, 73).

We have previously shown increased expression of certain members of the TGF-β superfamily by cultured human cardiac endothelial cells treated with doxorubicin (20) and that endothelial damage was mediated by the TGF-β pathway. Using an in vivo model of doxorubicin cardiomyopathy, we now report increased cardiac expression of the TGF-β isoforms. We have also shown enhanced Smad3 phosphorylation and nuclear translocation in response to a given concentration of TGF-β1 in doxorubicin-treated endothelial cells. Our transcriptomic analysis showed increased abundance of transcripts for TGF-β target genes and signaling intermediates in treated endothelial cells and demonstrated suppressed activity of the TGF-β pathway as a result of Smad3 knockdown. In addition to upregulation of the canonical Smad3-mediated pathway, transcriptomic analysis pointed to suppression of angiogenic factor signaling and induction of profibrotic and cell cycle arrest-related transcripts in this setting, effects that were ameliorated in Smad3-deficient cells. We further used an aortic explant model of angiogenesis to show that Smad3 mediates suppression of endothelial sprouting by doxorubicin.

Analysis of the cardiac endothelial transcriptome suggested that RNAs related to cytokine activation by doxorubicin were consistently suppressed in Smad3-deficient cells. Previous studies have suggested that biomarkers of inflammation were predictive of doxorubicin-induced cardiotoxicity in patients with breast cancer (74) whereas anti-inflammatory interventions alleviated doxorubicin toxicity in treated mice (75). These studies raise the possibility of chronic inflammation as a contributing factor in toxic effects by doxorubicin. As has been previously described by others, cardiac endothelial transcriptome is enriched in cytokine and cytokine receptor transcripts and develops enhanced inflammatory response to endotoxin, including increased expression of adhesion molecules and cytokine receptors, as compared with endothelia from other vascular beds (76, 77). Given the central role that endothelial cells play in inflammation, our results point to endothelial Smad3 as a critical proinflammatory effector in the setting of doxorubicin cardiomyopathy.

### Development of Doxorubicin Cardiomyopathy Is Smad3 Dependent

The consequences of Smad3 deletion have been previously examined in several animal models of cardiovascular diseases (21, 78–81), but to our knowledge, this is the first evaluation of the role of this transcription factor in cardiac injury by doxorubicin. In our model, we observed systolic dysfunction in wild-type mice after completion of a doxorubicin treatment regimen. In particular, we detected in these hearts a drastic decline in radial strain, a myocardial deformation index that correlated well with the histological and biochemical hallmarks of cardiomyopathy and has been useful in early detection of doxorubicin cardiomyopathy (82). Reduced myocardial strain is also observed in patients treated with doxorubicin and has a proven prognostic value (83, 84). Left ventricular dilation was also detected in hearts of the doxorubicin-treated wild-type mice, a type of cardiac remodeling that is often seen in treated patients. Both contractile decline and left ventricular remodeling were prevented in Smad3-knockout mice treated with doxorubicin. Involvement of Smad3 in upregulated inflammatory responses has been supported by several recent studies using such diverse models as ischemic nephropathy, hypertensive renal and cardiac remodeling, healing myocardial infarct, and orthotopic heart transplant rejection (21, 78, 85–87). In addition, Smad3 is known to function as a feed-forward TGF-β autoinduction factor that contributes to the increased cellular production and release of TGF-β in the inflamed tissue (88) to fuel chronic inflammatory response in hearts of doxorubicin-treated wild-type animals. This study has provided evidence that doxorubicin activates multiple cytokine and chemokine pathways in both cultured cardiac endothelial cells and mouse hearts in vivo, and such inflammatory response is mediated by Smad3. In particular, IL-6 levels were acutely elevated in doxorubicin-treated endothelial cultures and wild-type hearts, a response that was blunted in Smad3-deficient models. Inflammatory cytokines have been implicated in cardiac remodeling and pathophysiology of heart failure in both clinical and animal studies. Specifically, knocking out IL-6 attenuated cardiac dysfunction and cardiac remodeling in a mouse model of transverse aortic constriction (89). In a large cohort of patients with heart failure, elevated IL-6 levels were found in over 50% of patients and were associated with reduced left ventricular ejection fraction (LVEF) and poor clinical outcomes (90).

As suggested by transcriptomic analysis of both mouse and human cardiac endothelial cells, chronic inflammatory response to doxorubicin appears to be a component of a broader long-lasting endothelial reprogramming that also involves suppression of the VEGF pathway, cell cycle arrest, and senescence. Persistent expression of the senescence markers was previously detected in cardiac endothelial cells but not cardiomyocytes of mice treated with doxorubicin (17). The study demonstrated direct relation of this persistent senescent phenotype to doxorubicin toxicity. TGF-β, cytokine, and p38 mitogen-activated protein kinase pathways have been previously shown to contribute to development of the senescent phenotype (91, 92). Our analysis has shown that all these pathways are activated by doxorubicin but downregulated in Smad3-deficient endothelial cells. Endothelial cells are known to regulate cardiomyocyte contraction by secreting soluble factors that influence their function (93–95). In this regard, it is important to emphasize a so-called senescence-associated secretory phenotype that is defined as sustained highly elevated production of cytokines, chemokines, and growth factors by senescent cells. This phenomenon has been previously described in doxorubicin-treated cultured human endothelial cells (92). Therefore, both doxorubicin itself and inflammatory cytokines produced in treated hearts likely alter the endothelial secretome. As reported by Juni et al. (96), exposure of human cardiac microvascular endothelial cells to inflammatory cytokines impaired cardiomyocyte function by suppressing endothelial nitric oxide production. It is noteworthy in this regard that doxorubicin pretreatment impaired acetylcholine-induced dilation in atrial vessels, an endothelial nitric oxide-dependent phenomenon (97). Thus, by effectively suppressing cardiac inflammation in doxorubicin-treated hearts, Smad3 deficiency likely restores the endothelial cell secretome to better support cardiomyocyte function. In addition to nitric oxide, endothelium is known to produce other factors that support cardiac function and alleviate progression of heart failure, including neuregulins and apelin (94, 98–100). As shown in Ref. 95, endothelial production and release of these factors is mediated by VEGF signaling via VEGF receptor-2 (VEGFR2), a pathway that shows transcriptomic suppression by doxorubicin.

### Study Limitations

A global Smad3-knockout model did not allow us to dissect cell-autonomous effects of deletion of this transcription factor and therefore unequivocally attribute beneficial effects observed in doxorubicin-treated Smad3-deficient animals to a specific cell type. However, the notable accumulation of doxorubicin in endothelial cells does implicate these cells. It is also plausible that Smad3 deficiency in other cardiac cell types may have contributed to the observed amelioration of cardiomyopathy in treated animals. Although studies of Smad3 in myocytes are sparse, earlier reports have suggested a role of the TGF-β/Smad2/3 pathway in hypertrophic remodeling and induction of apoptosis of isolated cardiomyocytes (101, 102). Also, cardiomyocyte-specific Smad3 deletion can attenuate hypertrophic remodeling and systolic dysfunction in a mouse model of myocardial infarction (103). Thus, it remains a possibility that Smad3 is directly involved in cardiotoxic effects of doxorubicin on both endothelial cells and cardiomyocytes.

### Conclusions and Future Directions

To summarize, doxorubicin accumulates in cardiac endothelium of treated animals and enhances the TGF-β/Smad3 pathway and inflammation in both cultured endothelial cells and hearts of treated animals. Whereas doxorubicin caused dilated cardiomyopathy in wild-type animals, inflammation, contractile decline, and left ventricular remodeling were prevented in Smad3-deficient mice. This suggests that the sustained changes in endothelial gene expression and adverse cardiac remodeling and dysfunction induced by doxorubicin are Smad3 dependent. Further mechanistic studies are warranted to elucidate the processes that enable cardiomyopathic remodeling by Smad3 in this setting. Considering the profound effects of Smad3 deletion on doxorubicin-treated endothelial cells, their critical role in cardiac production cytokines, mounting tissue inflammation, and maintaining cardiomyocyte function, future studies using endothelium-specific Smad3-knockout models are warranted. Future efforts that focus on characterizing the endothelial secretome under these conditions appear worthwhile as well. As the established antitumor mechanisms of doxorubicin are not mediated by Smad3 (104), suppression of the pathway is not predicted to compromise the anticancer activity of this drug. Our study suggests the potential value of novel approaches to ameliorate doxorubicin cardiotoxicity that target activity of the Smad3 transcription factor.

## DATA AVAILABILITY

All RNA sequencing data were uploaded to the Gene Expression Omnibus (GEO) database (Accession No. GSE206743 and No. GSE206679).

## SUPPLEMENTAL DATA

10.6084/m9.figshare.21202964 Supplemental Figs. S1–S3: https://doi.org/10.6084/m9.figshare.21202964.

## GRANTS

This work was supported by National Heart, Lung, and Blood Institute Grant R15HL133873 (to E.A.K.).

## DISCLOSURES

No conflicts of interest, financial or otherwise, are declared by the authors.

## AUTHOR CONTRIBUTIONS

B.D. and E.A.K. conceived and designed research; M.S.C., S.T., M.G., J.H., J.S., Z.C., E.D., K.C., and E.A.K. performed experiments; M.S.C., S.T., K.S., M.G., J.H., Z.C., E.D., K.C., D.P.H., and E.A.K. analyzed data; M.S.C., K.S., D.P.H., and B.D. interpreted results of experiments; M.S.C., S.T., K.S., M.G., Z.C., and E.A.K. prepared figures; R.V.S. and E.A.K. drafted manuscript; D.P.H., R.V.S., B.D., and E.A.K. edited and revised manuscript; M.S.C., S.T., K.S., M.G., J.H., J.S., Z.C., E.D., K.C., D.P.H., R.V.S., B.D., and E.A.K. approved final version of manuscript.
